# Breastfeeding and coronavirus disease‐2019: Ad interim indications of the Italian Society of Neonatology endorsed by the Union of European Neonatal & Perinatal Societies

**DOI:** 10.1111/mcn.13010

**Published:** 2020-04-26

**Authors:** Riccardo Davanzo, Guido Moro, Fabrizio Sandri, Massimo Agosti, Corrado Moretti, Fabio Mosca

**Affiliations:** ^1^ Institute for Maternal and Child Health IRCCS “Burlo Garofolo” Trieste Italy; ^2^ Technical Panel on Breastfeeding Ministry of Health Rome Italy; ^3^ Human Milk Banking Association of Italy (AIBLUD) Milan Italy; ^4^ Neonatal Intensive Care Unit Maggiore Hospital Bologna Italy; ^5^ Woman and Child Department Ospedale Del Ponte Varese Italy; ^6^ President of Union of European Neonatal and Perinatal Societies; Emeritus Consultant in Pediatrics, Policlinico Umberto I Sapienza University Rome Italy; ^7^ President of Italian Society of Neonatology; Fondazione IRCCS Ca’ Granda Ospedale Maggiore Policlinico University of Milan Italy

**Keywords:** birth, breastfeeding, breastfeeding promotion, human milk, infectious disease, neonate

## Abstract

The recent COVID‐19 pandemic has spread to Italy with heavy consequences on public health and economics. Besides the possible consequences of COVID‐19 infection on a pregnant woman and the fetus, a major concern is related to the potential effect on neonatal outcome, the appropriate management of the mother–newborn dyad, and finally the compatibility of maternal COVID‐19 infection with breastfeeding. The Italian Society on Neonatology (SIN) after reviewing the limited scientific knowledge on the compatibility of breastfeeding in the COVID‐19 mother and the available statements from Health Care Organizations has issued the following indications that have been endorsed by the Union of European Neonatal & Perinatal Societies (UENPS). If a mother previously identified as COVID‐19 positive or under investigation for COVID‐19 is asymptomatic or paucisymptomatic at delivery, rooming‐in is feasible, and direct breastfeeding is advisable, under strict measures of infection control. On the contrary, when a mother with COVID‐19 is too sick to care for the newborn, the neonate will be managed separately and fed fresh expressed breast milk, with no need to pasteurize it, as human milk is not believed to be a vehicle of COVID‐19. We recognize that this guidance might be subject to change in the future when further knowledge will be acquired about the COVID‐19 pandemic, the perinatal transmission of SARS‐CoV‐2, and clinical characteristics of cases of neonatal COVID‐19.

**Key messages**

- According to SIN/EUNPS, an asymptomatic and paucisymptomatic COVID‐19 positive mother may breastfeed her neonate in a rooming‐in regimen, under strict measures of infection control.
- When a mother with COVID‐19 is too sick to care for the newborn, the neonate will be managed separately and fed fresh expressed breast milk with no need to pasteurize it.

## 1 INTRODUCTION

The recent pandemic caused by a novel coronavirus isolated in Wuhan (Hubei Region, China) at the end of 2019 (SARS‐CoV‐2)(Ashour, Elkhatib, Rahman, & Elshabrawy, 2020; Dong et al., 2020; Lai, Shih, Ko, Tang, & Hsueh, 2020; Sung, Lu, Xu, Sun, & Pan, 2020; Wu & McGoogan, 2020; Zhao et al., 2020) has recently struck Italy with heavy consequences on public health and economics (Remuzzi & Remuzzi, 2020). The 28th March 2020 Bulletin of the Italian Civil Protection reports 92,472 cases (Protezione Civile, 2020). Among other clinical and public health issues, a major concern is raised by COVID‐19 during pregnancy and the possible transmission of the infection from mother to child before, during, and after childbirth (ACOG, 2020; Rasmussen, Smulian, Lednicky, Wen, & Jamieson, 2020; CNGOF, 2020; Mullins, Evans, Viner, O'Brien, & Morris, 2020; Quiao, 2020). Particularly, the option of the joint management of mother and child after childbirth and the safety of breastfeeding are questioned. In order to deal with these issues, an expert panel of the Italian Society of Neonatology (SIN) conducted a review of published literature and developed the present consensus statement, endorsed by the Union of European Neonatal & Perinatal Societies (UENPS).

## 2 CURRENT KNOWLEDGE

The SARS‐CoV‐2 virus spreads mainly from person to person through close contact (0–2 m) and is transmitted by means of respiratory secretions (droplets) when an infected individual sneezes or coughs (CDC, 2020). The efficiency of SARS‐CoV‐2 transmission via the enteral route (Gu, Han, & Wang, 2020), through the conjunctival mucosa (Peng & Zhou, 2020), or after contact with contaminated environmental surfaces (Ong et al., 2020; van Doremalen et al., 2020) is still to be defined, but would seem less likely.

It is not yet established if COVID‐19 might have a transplacental transmission. Nevertheless, in a similar way to past SARS‐CoV‐1 (severe acute respiratory syndrome) and MERS‐CoV (Middle East respiratory syndrome coronavirus) epidemics, fet al and neonatal outcome (e.g.,prematurity) may depend more on the severity of the maternal infection and on concurrent obstetric diseases, rather than on the SARS‐CoV‐2 infection from the pregnant woman to the fetus.

In a retrospective study on nine women with COVID‐19 pneumonia during the third trimester of pregnancy, SARS‐CoV‐2 was not detected in the amniotic fluid, in cord blood, nor in mother's breast milk; moreover, the pharyngeal swab of six neonates was also shown to be negative for SARS‐CoV‐2 RNA by real‐time PCR (RT‐PCR‐RNA; Chen, Guo, et al., 2020). According to Chen et al., the placenta of COVID‐19 pregnant women who undergo a caesarean section (CS) had no pathological changes and was negative for SARS‐CoV‐2 RNA (Chen, Huang, et al., 2020). Two recent articles reported that three neonates of COVID‐10 mothers showed elevated IgM antibodies to SARS‐CoV‐2, raising the possibility of a COVID‐19 infection acquired in utero (Dong, Tian, & Yang, 2020; Zeng et al., 2020). Nevertheless, as all three neonates were tested negative for RT‐PCR‐RNA, more evidence is needed before confirming a risk of fet al COVID‐19 (Kimberlin & Stagno, 2020).

In conclusion, current knowledge does not support an intrauterine transmission of COVID‐19, similar to the SARS‐CoV during the 2002–2003 epidemic in Asia (Schwartz & Graham, 2020). Consequently, a neonatal COVID‐19 might be the result of a transmission acquired by the mother via the respiratory route in the post‐partum period rather than antenatally.

### 2.1 Neonatal COVID‐19

The possibility of a respiratory infection from common coronaviruses in the neonatal period and in the first year of life had already been observed prior to the current SARS‐CoV‐2 outbreak (van der Zalm et al., 2009; Jean, Quach, Yung, & Semret, 2013).

Zhu et al. (2020) has described a cohort of nine neonates whose mothers had suspected COVID‐19. Seven of the nine infants were born after a CS. No information on the type of feeding was provided. All neonates developed respiratory symptoms in the first week of life and received a clinical diagnosis of pneumonia, but their viral test from pharyngeal swabs proved to be negative, thus not corroborating the SARS‐CoV‐2 aetiology. Relatively few COVID‐19 neonates have been reported in the scientific literature during the Hubei epidemic (Cao, Chen, Chen, & Chiu, 2020).

A recent paper from *JAMA Pediatrics* reports that among 33neonates of COVID‐19 mothers, three presented respiratory symptoms in the first days of life and tested positive for COVID‐19 on Day 2after childbirth (Zeng, Xia, & Yuan, 2020). The only seriously ill neonate in this case series was a 31‐week gestational age preterm infant.

Although we cannot ignore that a COVID‐19 infant might develop a respiratory failure and be admitted to an NICU (Paediatric Committee, 2020; De Luca, 2020; Wang, Qi, Bao, Li & Shi, 2020; Wang, Gou, et al., 2020), to date, reported cases are usually mild, with a favourable outcome (Cao et al., 2020; Lu & Shi, 2020). Nevertheless, a neonate delivered by a COVID‐19 mother requires a complex hospital organization with the provision of an isolated room for mothers and/or neonates and firm implementation of the protective measures against contagion for health professionals (Wang, Shi, et al., 2020).

Summarizing, based on the available limited literature, neonatal COVID‐19 (1) appears to have a horizontal transmission and (2) seems to be paucisymptomatic or asymptomatic compared with older age groups.

### 2.2 COVID‐19 infection in the first year of life and beyond

Wei et al. (2020) have described a series of nine COVID‐19‐positive infants, aged 56 days to 11 months, with a history of intrafamiliar transmission and presenting with fever, cough, respiratory secretions, and rhinitis. Their general health conditions were fair, and none required intensivecare.

On the basis of current evidence, paediatric COVID‐19 appears to be mild or asymptomatic (Cao et al., 2020; Chan et al., 2020) similarly to the 2002–2003 SARS‐CoV epidemic (Shek et al., 2003; Li, 2005). This is particularly true for COVID‐19 in the first year of life and generally under 10 years of age (Wei et al., 2020; Zhang et al., 2020).

A recent study from China on 2,143 children with respiratory symptoms included data on 379 infants; among infants, only a minority (22.7%) tested positive to COVID‐19. Although 10.6% of infants were reported to be severe, the authors also note that greater severity, over all ages, was found in not confirmed COVID‐19 children, possibly due to different aetiological agents (Dong et al., 2020). This information should be carefully considered as policies are being instituted requiring maternal separation and precluding breastfeeding on the basis that COVID‐19 can cause severe illness. On the contrary, separation might expose infants to a greater risk of infection with other pathogens that are more likely to cause serious illness.

## 3 PROMOTION OF BREASTFEEDING AND INTEGRATION WITH INFECTION CONTROL MEASURES

Breastfeeding improves the health of mother and child, implies benefits for families, and has a positive social and economic impact (Davanzo, Romagnoli, & Corsello, 2015; Rollins et al., 2016).

Based on current scientific knowledge, the breast milk of a COVID‐19 mother cannot be considered a transmission vehicle, in a similar way that other known respiratory viral infections cannot be (World Health Organization [WHO], 2020).

The precautionary indication to COVID‐19 mothers for not breastfeeding has been proposed by some authors (Favre et al., 2020) without sound evidence and clearly ignores the importance of breastfeeding (Binns, Lee, & Low, 2016). On the contrary, the current COVID‐19 pandemic leads us to combine the promotion of breastfeeding with correct infection control measures, in order to limit the contagion by droplets and by contact with the respiratory secretions of infected patients (including mothers having just given birth; Centers for Disease Control and Prevention [CDCb], 2020).

We can speculate that, similar to the 2002–2003 SARS‐CoV epidemic (Robertson et al., 2004), specific SARS‐CoV‐2 antibodies pass via the breast milk from the COVID‐19 mother to the infant within a few days after the onset of the disease, thus possibly modulating the clinical expression of the infant's infection.

An approach involving the routine separation of the newborn from the COVID‐19 mother not only interferes with the mother–child relationship (WHO & United Nations Children's Fund [UNICEF], 2018) but might also be acting too late for a contagion that has already occurred in the presymptomatic phase.

## 4 CURRENTLY AVAILABLE DIRECTIONS ON THE PREVENTION OF MOTHER TO CHILD TRANSMISSION

### 4.1 Chinese Pediatrics COVID‐19 Working Group

Doctors who recently have dealt with the COVID‐19 epidemic in China suggest infant feeding with formula or possibly donor breast milk (Wang, Shi, et al., 2020). The authors do not provide specific reasons for this choice. In the recommendation of the Chinese experts, presumably unbalanced on the side of caution, there is no information of any overall assessment of the risks of infection compared with those of not breastfeeding.

### 4.2 World Health Organization

A woman with suspected, probable, or confirmed COVID‐19 can practice skin to skin contact in the delivery room and exclusively breastfeed her child (WHO, 2020). If maternal general health conditions impede direct breastfeeding, she should be encouraged and supported to express breast milk and feed it to her child. A COVID‐19 mother must always follow the infection prevention measures. Moreover, the environmental surfaces touched by the mother must be regularly cleaned and disinfected.

### 4.3 United Nations Children's Fund

This agency does not consider the option of separating mother and neonate and suggests to continue breastfeeding, with the concurrent adoption of hygiene measures to reduce the possible transmission of the COVID‐19 from mother to neonate (UNICEF, 2020).

### 4.4 Centers for Disease Control and Prevention

According to the CDC (CDCa, 2020),

- If the mother is under investigation or tests positive for COVID‐19, the option of ensuring mothers and neonates cared for in separate rooms should be considered as the first choice. We must observe that the indication by the CDC on the desirable separation of mothers and neonates is not based on an analysis of the impact that this might have on breastfeeding nor the likelihood that this option might result in formula feeding. The risks and benefits of this separation and the information on the consequences of not starting, continuing, or suspending breastfeeding should be shared with the family and with health care professionals.
- If a breastfeeding mother and her newborn infant are managed jointly, measures aimed at preventing the transmission of the viral infection should be put in place: avoid kissing the neonate, protect him from adult coughing and respiratory secretions (wear a mask during feeding and intimate contact with the baby), wash hands, in particular, before feeding, suspend visits. If the child stays in hospital with the mother in a rooming‐in regimen, he will be put to sleep in his cradle at a distance of at least 2 m from the ill mother; moreover, the use of a physical barrier, such as a curtain between the mother and newborn, may be appropriate.

### 4.5 Royal College of Obstetricians and Gynecologists

The Royal College of Obstetricians and Gynecologists (RCOG) is very clear in advising that mothers and babies should be kept together and consequently questions the Chinese recommendation to routinely separate the newborn from the COVID‐19 mother. According to the RCOG, mother and neonate separation should be justified by the poor health conditions of the mother or by the need to provide therapies to the newborn (RCOG, 2020). Moreover, the RCOG believes that breastfeeding should be recommended, given that the related benefits for the newborn outweigh the potential risks.

### 4.6 International Society of Ultrasound in Obstetrics and Gynecology (ISUOG)

Rooming‐in and breastfeeding are options as far as a mother with COVID‐19 is not severely affected (Poon et al., 2020).

### 4.7 Italian National Institute of Health

In the light of current available scientific data and the protective potential of breast milk, a woman with suspected or confirmed COVID‐19, under favourable clinical conditions and according to her desire, should start and continue to breastfeed, directly to the breast or using expressed breast milk (Italian National Institute of Health [ISS], 2020). To reduce the risk of transmission to the child, preventive procedures, such as hand cleaning and the use of a face mask during feeds, are advisable.

### 4.8 Academy of Breastfeeding Medicine

The hospital management of mothers suspected of or with confirmed COVID‐19 must foresee two options: rooming‐in or separation between mother and neonate. The choice mainly depends on the general health conditions of the woman and must necessarily involve also mother and family. As an alternative to sucking directly to the breast, expressing breast milk should be considered a safe procedure (Academy of Breastfeeding Medicine [ABM], 2020).

## 5 PROVISIONAL INDICATIONS OF SIN AND UENPS ON THE MANAGEMENT OF MOTHER AND NEONATE DURING THE COVID‐19 PANDEMIC

The provisional directions of SIN and UENPS, coherent with the recommendations made by WHO, UNICEF, ISS, IUOG, RCOG, and ABM, are summarized in Table 1.

**FIGURE 1:**
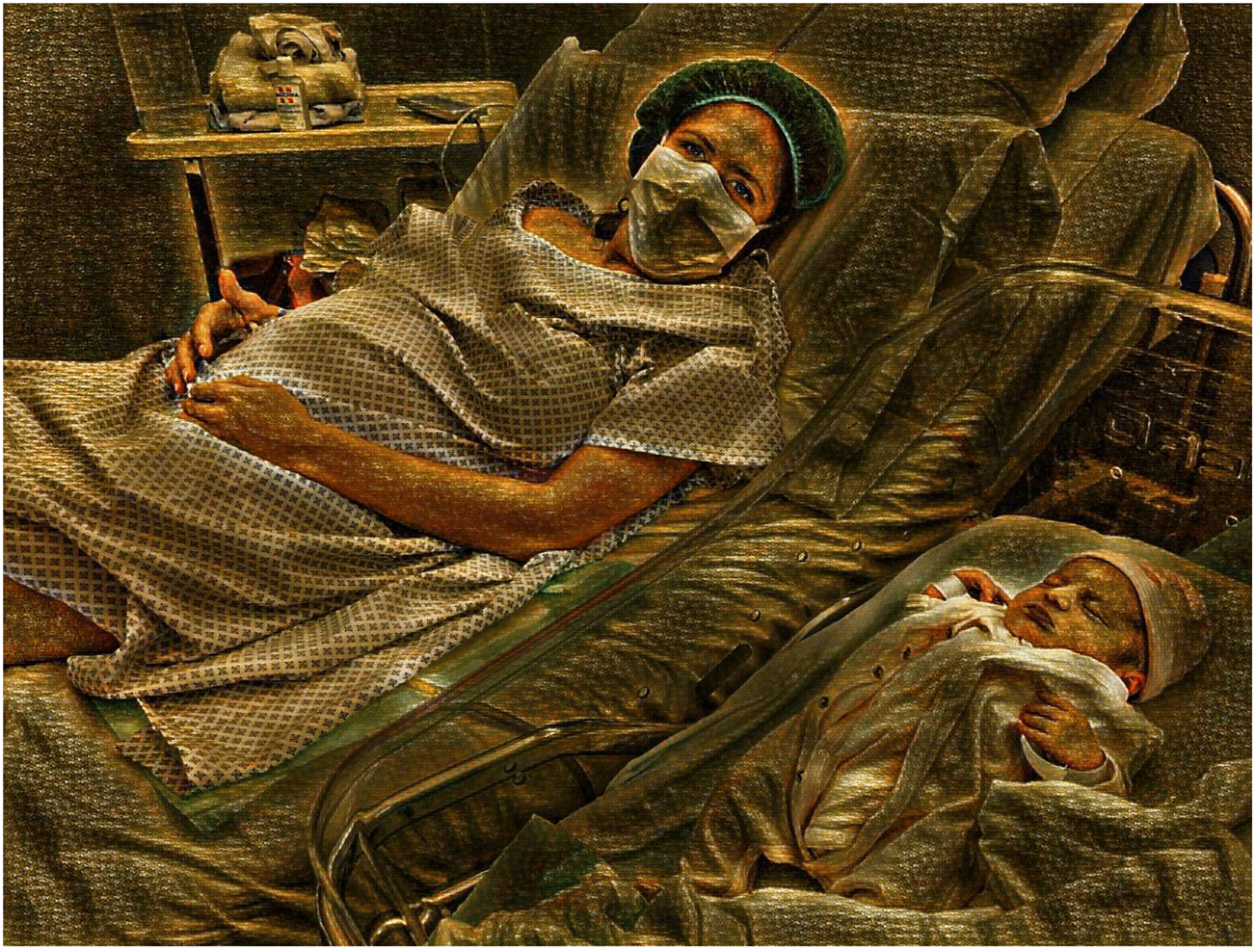
Rooming‐in of a mother with COVID‐19 together with her neonate. This regimen is applied in case of a asymptomatic or paucisymptomatic mother where droplets passage via coughing is not a concern and the mother is able to take care for the neonate. Graphic processing by Marco Davanzo from an original photo taken at Clinica Mangiagalli, Milan, on March 2020 [Colour figure can be viewed at wileyonlinelibrary.com]

- Whenever possible, the preferred option is the joint management of the mother and her infant, in order to facilitate their interaction and the beginning of breastfeeding (Figure 1). This choice is defined by the good health status of both the mother and her neonate. Usually, the mother is asymptomatic or paucisymptomatic, previously identified as positive to COVID‐19 or under investigation for COVID‐19.
- A mother affected by severe respiratory infection (with fever, coughing, and respiratory secretions) and too sick to care for her neonate should be temporarily separated, pending the result of RT‐PCR‐RNA test for COVID‐19. If the test is positive, mother and child continue to be managed separately; if the test is negative, rooming‐in for the dyad is applicable as far as the mother is ready to take care of the neonate.
- At present, the decision about whether or not to separate mother and neonate must be individualized, taking into account the informed consent of the mother, the hospital logistics, and possibly the local epidemiological situation of current COVID‐19 pandemic.
- In case of separation of mother and neonate, the routine use of breast milk substitutes should be avoided; we rather recommend implementing the expression, transportation, and administration of the fresh mother's milk to the neonate.
- Expressed breast milk should not be pasteurized, as it is not believed to be a vehicle of infection, even if it contains SARS‐CoV‐2. Moreover, pasteurization reduces the biological and immunological value of human milk.
- A premature or sick newborn infant requiring intensive care should be admitted in an isolated area of the Neonatal Intensive Care Unit (NICU) and cared for by a skilled neonatal team wearing an appropriate personal protective equipment (PPE). In this setting, the expressed breast milk of a COVID‐19 mother should be transported, processed, and administered in accordance to the NICU specific protocol. Present knowledge does not support routine pasteurization of the COVID‐19 mother' milk prior to administration to a preterm or sick newborn infant.
- In case of a COVID‐19 mother, strict hygiene measures should be adopted to prevent the possible transmission of the infection by droplets or by contact with respiratory secretions.
- In detail, the room should be isolated, not allowing visits of relatives and friends. The baby's cradle should be placed at a distance of 2 m from the mother's head, and a room divider or a curtain in between mother and neonate can also be used. Certainly, these measures, given the distance and separation from the neonate, will make it more difficult for mothers to feed their babies, particularly after a CS in the absence of visitors. Although COVID‐19 mothers will rely on hospital staff, this setting might have a negative impact on breastfeeding.
- Moreover, the mother should carefully wash her hands and wear a surgical face mask during breastfeeds and intimate contact with the newborn. There is no need for the mother to use FFP2 or FFP3 face mask.
- Lastly, the other hospitalized patients and the health care personnel should also be protected.
- The compatibility of breastfeeding with drugs eventually administered to a mother with COVID‐19 should be assessed on a case‐by‐case basis.
- Hospital discharge of a paucisymptomatic COVID‐19 mother together with a healthy SARS‐CoV‐2‐negative neonate should be done appropriately. Going home as early as 48 h after childbirth might be an option only in cases of hospital overload. In most cases, 1‐week hospital stay for surveillance of the newborn and repetition of the pharyngeal swab for SARS‐CoV‐2 at discharge is preferable.
- At home, according to the organization of the Italian National Health Care System, the neonate is in charge of the family paediatrician. The mother may continue to breastfeed and/or to express breast milk, depending on her health conditions and desire.
- Due to the risk of spreading COVID‐19 to ambulatory staff and patients/families, face‐to‐face meetings should be replaced as far as possible by telephone consultation and/or telemedicine to allow visual clinical assessment of the neonate, to guide parents for infants weighing and to provide lactation advice.
- At 14 days after discharge, the infant should receive a check‐up and a pharyngeal swab for SARS‐CoV‐2.
- Neonatal COVID‐19 follow‐up can be stopped on the 28th day after discharge, if the pharyngeal swab for SARS‐CoV‐2 is confirmed negative.

**TABLE 1:** Indications on the management of mother and neonate in the perinatal period

Health status of the mother	Pharyngeal swab for COVID‐19 on the mother	Pharyngeal swab for COVID‐19 on the neonate	Isolation of the mother^a^	Management of the neonate during hospital stay^a^	Advice on direct breastfeeding	Preventive measures for mother–neonate transmission^c^
Asymptomatic or paucisymptomatic mother known to be COVID‐19 positive	Already done	Yes	Yes, in an isolated and dedicated area of post‐partum ward	In a rooming‐in regimen, in an isolated and dedicated area of post‐partum ward	Yes	Yes
COVID‐19 paucisymptomatic mother under investigation	Yes	Only if maternal test is positive	Yes, in an isolated and dedicated area of post‐partum ward, pending result of the lab test	In a rooming‐in regimen, in an isolated and dedicated area of post‐partum ward, at least until the result of the lab test	Yes	Yes
Mother with symptoms of respiratory infection (fever, cough, and secretions) and too sick to care for the newborn, COVID‐19 positive or under investigation	Yes or already being done	Only if maternal test is positive	Yes, in a dedicated and isolated area of post‐partum ward, pending result of the lab test	Neonate isolated and separated from the mother, at least until the result of the lab test. He is placed in a dedicated and isolated area in the Neonatology Unit (if asymptomatic) or in the NICU (if symptomatic; e.g. with respiratory disease)	No; use of expressed milk.^b^ pasteurization not recommended	Yes

a In addition, adequate protection measures on the part of health care personnel, according to the indication of the Ministry of Health of Italy.

b Mothers fresh milk should be expressed with a dedicated manual or electrical breast pump. The mother should always wash her hands before and after touching bottles and all breast pump parts, following recommendations for proper washing of the breast pump after each use.

c Room divider or curtain, surgical face mask for the mother during breastfeeds and intimate contact with the newborn, careful washing of hands, placing the baby's cradle at a distance of 2 m from the mother's head, and no visits of relatives and friends. No need of the mother to use FFP2 or FFP3 face mask (Radonovich, Simberkoff, & Perl, 2019; UENPS, 2020).

## 6 CONCLUSION

This document has been proposed by SIN and UENPS to provide maternity hospitals with management guidance. Given the current pandemic, the authors aimed to conjugate, as far as possible, an appropriate COVID‐19 infection control with the promotion of breastfeeding and the initial mother–infant relationship after childbirth. We recognize that this guidance might be subject to change in the future when further knowledge will be acquired about the COVID‐19 pandemic, its perinatal transmission, and clinical characteristics of cases of neonatal SARS‐CoV‐2 infection.

## CONFLICTS OF INTEREST

The authors declare that they have no conflicts of interest.

## CONTRIBUTIONS

RD and FM conceived the manuscript. RD and FM wrote the first draft of the manuscript. GM, FS, MA, and CM revised the manuscript and provided critical advice.

## NOTICE

This is the updated version of a document firstly disseminated on 28 February 2020. The present version takes into account the scientific data available as of 28 March 2020. The guidance given is subject to change in the future with the acquisition of further knowledge about the Coronavirus Disease‐2019 (COVID‐19) pandemic, its perinatal transmission, and clinical characteristics of cases of neonatal COVID‐19.

## GLOSSARY

We would clarify the meanings given to the terms SARS‐CoV‐2 and COVID‐19 in this document. The term SARS‐CoV‐2 is taken to mean the viral causative agent responsible for SARS, also called 2019 Wuhan novel coronavirus. The term SARS is the acronym for severe acute respiratory syndrome caused by coronavirus. COVID‐19 means the Coronavirus Disease identified at the end of 2019 in the Hubei Region, China.
